# Distinct Tissue Localization and Transcriptional Profiles of Shiga Toxin‐Producing *Escherichia coli* With and Without the Locus of Enterocyte Effacement at the Bovine Recto‐Anal Junction

**DOI:** 10.1155/ijm/2576911

**Published:** 2026-08-29

**Authors:** Diego Méndez, José T. Cartajena, Jacqueline Flores, Fernanda Quintanilla, Jorge Toledo, Pablo Cruz, Jessica Dorner, Beatriz Escobar, María Paz Ríos, Cristóbal Lavanderos, Vicente Ferrer, Paz Rojas, Daniela Luna, Lisette Lapierre, Indira T. Kudva, Víctor Martínez, Nicolás Galarce

**Affiliations:** ^1^ Department of Animal Science, Faculty of Veterinary and Animal Sciences, University of Chile, Santiago, Chile, uchile.cl; ^2^ Department of Animal Preventive Medicine, Faculty of Veterinary and Animal Sciences, University of Chile, Santiago, Chile, uchile.cl; ^3^ Master’s Program in Animal and Veterinary Sciences, Faculty of Veterinary and Animal Sciences, University of Chile, Santiago, Chile, uchile.cl; ^4^ Department of Health Sciences, University of Aysén, Coyhaique, Chile, uaysen.cl; ^5^ Department of Medical Technology, Faculty of Medicine, University of Chile, Santiago, Chile, uchile.cl; ^6^ CAMER Slaughterhouse, Santiago, Chile; ^7^ Food Safety and Enteric Pathogens Research Unit, National Animal Disease Center, Agricultural Research Service, United States Department of Agriculture, Ames, USA, usda.gov

**Keywords:** adhesion, cattle, colonization, gene expression, recto-anal junction, Shiga toxin-producing *Escherichia coli*

## Abstract

Shiga toxin‐producing *Escherichia coli* (STEC) colonizes the bovine recto‐anal junction (RAJ), a key step in STEC pathogenesis in cattle, associated with animal disease, persistence, fecal shedding, carcass contamination, and zoonotic transmission. However, early adhesion mechanisms at the bovine RAJ remain insufficiently characterized, particularly among serotypes lacking the Locus of Enterocyte Effacement (LEE), referred to as LEE‐negative strains. This study investigated strain‐dependent epithelial interaction patterns at the bovine RAJ using complementary *in vitro*, *ex vivo*, and transcriptional approaches. Four representative strains were selected for functional assays, including two LEE‐positive strains (O157:H7 and O26:H11) and two LEE‐negative strains (O113:H21 and O130:H11). All strains adhered to primary bovine RAJ squamous epithelial cells, although no significant quantitative differences were detected among strains. In RAJ explants, however, LEE‐positive strains showed epithelial association extending beyond the superficial layer, with immunolabeling distributed across the epithelial regions, whereas LEE‐negative strains showed a predominantly superficial distribution. This pattern, not previously described in bovine RAJ explants, expands the current view of STEC colonization as a uniformly superficial epithelial process. Analysis of selected adhesion‐related genes revealed strain‐dependent transcriptional responses to the RAJ environment. LEE‐positive strains showed higher expression of *eae*, together with increased expression of *iha*, *csgD*, and *pgaA*. In contrast, LEE‐negative strains showed lower expression of these genes and consistent expression of *saa*, supporting the existence of distinct epithelial interaction strategies that may reflect strain‐specific behaviors among circulating LEE‐positive and LEE‐negative isolates. Overall, these findings demonstrate distinct epithelial interaction and transcriptional profiles among STEC strains and show that physiologically relevant tissue models can reveal serotype‐dependent colonization patterns not fully captured in simplified cell systems and may support the identification of potential targets for vaccine‐based intervention strategies in cattle.

## 1. Introduction

Shiga toxin‐producing *Escherichia coli* (STEC) is an important zoonotic pathogen for which cattle represent the main reservoir. In humans, STEC infection can cause clinical outcomes ranging from diarrhea to hemorrhagic colitis and hemolytic uremic syndrome [1]. Outbreaks have been associated mainly with contaminated beef, fresh produce, water, and dairy products, with bovine feces representing an important source of contamination [2]. The public health relevance of STEC is reflected in US food safety regulations, as several serogroups, including O26 and O157, are considered adulterants in raw beef products [3].

In addition to its zoonotic importance, STEC may also contribute to enteric disease in calves [4–6]. However, adult cattle often remain clinically healthy while carrying STEC for prolonged periods, thereby acting as persistent reservoirs that facilitate within‐herd transmission and contamination of the food chain [7]. STEC detection in adult cattle is highly variable worldwide, with reported prevalence values reaching up to 90% [7–9]. This combination of asymptomatic carriage, fecal shedding, and cross‐contamination during slaughter makes bovine colonization a critical step in STEC persistence and transmission within cattle production systems.

In cattle, STEC transmission occurs mainly through the fecal–oral route, and the recto‐anal junction (RAJ) is considered a key site for bacterial adhesion, persistence, and long‐term colonization. This region includes follicle‐associated epithelium and recto‐anal junction squamous epithelial (RSE) cells, both of which can support STEC attachment [10]. Because colonization at the RAJ is closely linked to persistence in the bovine host, early adhesion events at this site are regarded as an important component of STEC pathogenesis in cattle. In strains harboring the Locus of Enterocyte Effacement (LEE), referred to as LEE‐positive strains, adhesion is typically associated with intimin (Eae) and the type III secretion system (T3SS), whereas strains lacking this locus, referred to as LEE‐negative strains, appear to rely on alternative adhesins, including Saa, Iha, and other factors associated with epithelial colonization [11, 12]. Nevertheless, the mechanisms underlying successful colonization at the bovine RAJ remain incompletely understood.

Bovine RAJ‐based models provide a biologically relevant framework for studying STEC adherence under conditions that better resemble the natural host environment [10, 13]. However, most functional studies using these models have focused on a limited number of serotypes, particularly O157:H7, and comparative data across LEE‐positive and LEE‐negative lineages remain scarce. Consequently, serotypes frequently detected in cattle, such as O130:H11 and O113:H21, are still underrepresented despite their relevance to bovine colonization and public health [14, 15]. In addition, transcriptional responses during STEC interaction with bovine RAJ explants have been only sparsely investigated. These gaps limit our understanding of whether distinct STEC lineages display different early colonization strategies at the bovine RAJ and hamper the development of targeted pre‐harvest control strategies.

In this study, we investigated early colonization‐related traits of LEE‐positive and LEE‐negative STEC strains using bovine RSE cells and RAJ explants, to determine whether distinct STEC lineages display different epithelial interaction patterns in physiologically relevant bovine models. By integrating *in vitro*, *ex vivo*, and transcriptional approaches, we sought to identify strain‐dependent differences associated with early adhesion at the bovine RAJ. A better understanding of STEC colonization mechanisms in cattle may help identify targets for pre‐harvest interventions aimed at reducing bovine carriage, food‐chain contamination, and the associated risk to animal and human health within a One Health context.

## 2. Materials and Methods

### 2.1. STEC Strains

Four STEC strains were selected from our collection representing distinct adhesion‐related phenotypic and genomic profiles. These included two LEE‐negative strains isolated from adult cattle in Chile, O113:H21 and O130:H11. In addition, two human clinical strains (O157:H7 and O26:H11) obtained from the Chilean National Institute of Public Health were included for comparison with the bovine isolates. The genomes of all strains were previously sequenced [14], and the corresponding sequences are available in GenBank under BioProject PRJNA656305. Relevant phenotypic characteristics previously determined using standardized *in vitro* assays, together with selected genotypic characteristics, are summarized in Table S1. The *E. coli* K‐12 (Microbiologics) strain was used as a nonpathogenic control when required.

### 2.2. Adhesion to RSE Cells

The four STEC strains were evaluated for their ability to attach to bovine RSE cells *in vitro*. *E. coli* K‐12 was used as a non‐pathogenic control. RSE cells were isolated from RAJ tissues collected at abattoirs following established protocols [10, 16]. Briefly, RAJ tissues were transported on ice in Dulbecco’s Modified Eagle Medium without glucose (DMEM‐NG; Gibco) supplemented with 2.5% fetal bovine serum (FBS, Gibco), antibiotics, and antimycotics (100 *μ*g/mL streptomycin, 100 U/mL penicillin, 50 *μ*g/mL gentamicin, and 2.5 *μ*g/mL amphotericin B), referred to as DMEM‐NG(+). In the laboratory, tissues were trimmed, washed, and incubated in fresh supplemented medium at 4°C for 24 h. RSE cells were then gently scraped, collected, and their viability was assessed by Trypan blue exclusion.

For adhesion assays, thawed RSE cells were adjusted to 10^5^ cells/mL in low‐glucose DMEM (DMEM‐LG; Gibco) and co‐incubated with each strain at a multiplicity of infection of 10:1 (bacteria:cell) for 4 h at 37°C with shaking (110 rpm) [17]. Bacterial suspensions were standardized to 10^6^ CFU/mL, as estimated using a DensiCheck Plus instrument (bioMérieux, France) and confirmed by serial dilution plating. After incubation, suspensions were centrifuged, washed, and spotted onto poly‐L‐lysine‐coated glass slides (Sigma‐Aldrich), fixed, and immunostained. STEC strains were detected using a goat polyclonal anti‐*E. coli* primary antibody (Invitrogen, MA, United States; catalog no. PA173032; lot no. ZG4414977) followed by Alexa Fluor 647‐labeled donkey anti‐goat IgG secondary antibody (Invitrogen). Epithelial cells were labeled with mouse anti‐pan cytokeratin antibody (BioLegend, CA, United States) and Alexa Fluor 488‐labeled goat anti‐mouse IgG (Invitrogen), while nuclei were stained with 4 ^′^,6 ^′^‐diamidino‐2‐phenylindole (DAPI, Invitrogen). Uninfected RSE cells served as negative controls. Each experiment included three biological replicates (cells obtained from different animals), and each biological replicate was analyzed in three technical replicates.

Images were acquired using a Leica TCS SP8 confocal microscope (Leica Microsystems). Confocal image stacks were deconvolved using AutoQuant X3 (Media Cybernetics) and analyzed in Imaris v10 (Bitplane) for 3D segmentation and quantification. Epithelial cells were segmented using the Cells module, and bacterial cells were identified using the Spots Detector tool. Bacteria associated with the segmented epithelial surface were considered adherent. Results were expressed as the mean number of adherent bacteria per cell across three biological replicates, each analyzed in triplicate.

Attachment patterns were evaluated qualitatively as aggregative, diffuse, or nonadherent [18], and bacterial adhesion counts were statistically analyzed in GraphPad Prism v10.6.1 (GraphPad Software) using the Kruskal–Wallis test followed by Dunn’s multiple‐comparison test. Differences were considered significant at *p* < 0.05.

### 2.3. Adhesion Assay in RAJ Explants

Adhesion to bovine RAJ explants was assessed using the same four STEC strains, following the protocol reported by Kudva et al. [13]. Briefly, the squamous epithelium of the bovine RAJ was collected at abattoirs and transported on ice in DMEM‐NG(+) (Gibco). In the laboratory, tissues were rinsed, the muscular layer was removed, and pieces of approximately 2 × 4 cm were placed mucosal side up on sterile Whatman filters (Sigma‐Aldrich) supported by a sponge soaked in DMEM‐high glucose (Gibco) supplemented with 10% FBS (Gibco) in six‐well plates, to maintain tissue viability from the basal surface. Tissue edges were secured with sterile pins and sealed with 3% agarose (w/v) to restrict the inoculum to the mucosal surface. Explants were immediately inoculated with 2 mL of standardized overnight STEC cultures in DMEM‐LG (10^8^ CFU/mL) and incubated at 37°C with shaking (100 rpm) for 4 h. Explant viability was assessed by Trypan blue exclusion (Gibco) at baseline and post‐infection [19]. Three biological replicates (explants from different animals), three technical replicates per explant, and three uninoculated control explants were included.

After incubation, explants were washed with sterile PBS (Gibco), fixed in 10% neutral‐buffered formalin (Thermo Fisher Scientific) for 24 h, and paraffin‐embedded. Sections (5 *μ*m), obtained from the central mucosal region of each explant away from the pinned and agarose‐sealed margins, were mounted on poly‐L‐lysine‐coated slides (Sigma‐Aldrich), dried overnight at 37°C, deparaffinized, and stained with hematoxylin‐eosin (H/E, Sigma‐Aldrich) [20]. For immunohistochemical (IHC) detection of STEC, antigen retrieval was performed in 10 mM citrate buffer (pH 6.0) at 95°C for 20 min. After cooling, nonspecific binding was blocked with 5% bovine serum albumin in PBS (Gibco) for 1 h, and slides were incubated overnight at 4°C with goat anti‐*E. coli* primary antibody (Invitrogen). After PBS washes, sections were incubated with the EnVision + Dual Link System‐HRP (DAB+) (Dako‐Agilent) for 30 min, and the signal was developed using 3,3 ^′^‐diaminobenzidine (DAB). Counterstaining was performed with Mayer’s hematoxylin, followed by dehydration and mounting. Negative controls were processed without bacterial inocula.

Images were captured using a Leica DM500 light microscope equipped with a Leica ICC50 W digital camera. Representative fields were photographed at 40× and 100× magnification. Positive immunolabeling was identified as brown‐gold cytoplasmic or membranous staining within epithelial cells [21].

To compare the distribution of bacterial immunolabeling across the epithelium, DAB signal was quantified using a standardized digital image analysis workflow in Fiji (ImageJ v2.18.0). Hematoxylin and DAB signals were separated by color deconvolution using the H DAB vector, and all subsequent analyses were performed on the DAB channel. Based on the histological architecture of the epithelium, three regions of interest (ROIs) corresponding to the basal, middle, and apical portions of the epithelial thickness were manually delineated for each image using the ROI Manager. Thresholding of the DAB signal was calibrated using noninfected negative control sections processed in parallel. For each ROI, the percentage of DAB‐positive area (DAB%) was quantified. DAB% was used as the primary quantitative outcome to compare bacterial immunolabeling among STEC strains within each epithelial region. Differences in DAB% among STEC strains were evaluated separately within each epithelial region (apical, middle, and basal) using one‐way ANOVA, followed by Tukey’s multiple‐comparisons test in GraphPad Prism v10.6.0 (GraphPad Software). Statistical significance was set at *p* < 0.05.

### 2.4. Quantification of Adhesion‐Related Gene Expression During RAJ Explant Infection

Expression of six selected adhesion‐related genes was quantified in the same four STEC strains to assess transcriptional changes underpinning bovine RAJ colonization. The evaluated genes were *csgD*, *pgaA*, *eae*, *iha*, *fliC*, and *saa*, selected based on their reported association with adhesion and colonization traits [22–26]. Primer sequences are provided in Table S2. Gene expression was compared between bacteria grown in DMEM‐LG and bacteria recovered from infected RAJ explants, following the protocol described by Genís et al. [27].

For broth‐grown controls, a single colony of each strain grown on salt‐free Luria Bertani agar (Oxoid) was inoculated into DMEM‐LG (Gibco) and incubated statically at 37°C overnight. Cultures were then diluted 1:5 in fresh DMEM‐LG and incubated for an additional 18 h at 37°C to reach stationary phase, after which 1 mL of each culture was stabilized in 1 mL RNAlater (Thermo Fisher Scientific). In parallel, RAJ explants were infected with standardized STEC cultures prepared in DMEM‐LG, as described above. After infection, explants were washed twice in PBS to remove nonadherent bacteria and immersed in RNAlater.

Total RNA was extracted using TRIzol Reagent (Thermo Fisher Scientific) according to the manufacturer’s instructions. RNA concentration was determined using the Qubit RNA BR Assay Kit, integrity was assessed with a Fragment Analyzer (Agilent), and purity was evaluated using an Epoch microplate spectrophotometer (Biotek Instruments). Samples were treated with DNase I (Invitrogen), and cDNA was synthesized using SuperScript First‐Strand (Invitrogen). cDNA concentration was determined using the Qubit ssDNA Assay Kit (Invitrogen).

qPCR reactions were performed in triplicate in a final volume of 10 *μ*L containing KAPA SYBR FAST Master Mix (2×; Roche), 0.2 *μ*L of each primer, 1 *μ*L of cDNA (10 ng), and nuclease‐free water. Amplification was carried out on a Rotor‐Gene Q thermocycler (Qiagen) with an initial denaturation at 95°C for 3 min, followed by 40 cycles of 95°C for 3 s and 60°C for 20 s. *gapA* (Table S2) was used as reference gene [16], and relative expression was calculated using the *ΔΔ*Ct method [28].

Differences in relative expression among strains were assessed by comparing *ΔΔ*Ct values using one‐way ANOVA followed by Tukey’s multiple‐comparison test when the gene was present in three or more strains, or by an unpaired Student’s *t*‐test when the gene was present in only two strains. Statistical analyses were performed in GraphPad Prism v10.6.0 (GraphPad Software), and differences were considered significant at *p* <0.05.

### 2.5. Use of Artificial Intelligence Generated Content

During the preparation of this work the authors used ChatGPT by OpenAI to improve readability and language. Authors reviewed and edited all content as needed. All scientific concepts, conclusions, and interpretations were developed by the authors. The authors take full responsibility for the content of the published article.

## 3. Results

### 3.1. Adherence to RSE Cells

**Figure 1:**
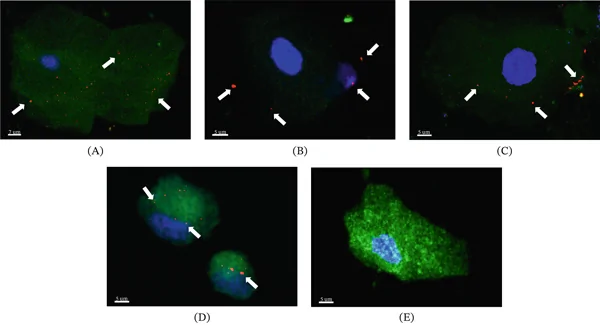
Immunofluorescence visualization of STEC adherence to primary bovine RSE cells. RSE cells are shown in green, bacteria in red, and nuclei in blue. Image brightness and contrast were adjusted uniformly to facilitate visualization of adherent bacteria. Arrows indicate representative adherent bacteria. Panels show adherence for (A) O157:H7, (B) O26:H11, (C) O113:H21, (D) O130:H11, and (E) noninfected control.

Adherence patterns were not associated with LEE status. O157:H7 was the only strain that displayed an aggregative adherence pattern, whereas the other three strains showed diffuse adherence, with occasional small aggregates observed in O130:H11 (Figure 1A–D). However, no statistically significant differences among strains were detected in the mean number of bacteria attached per cell (Table S3). As expected, no bacteria were detected in uninfected control cells (Figure 1E).

### 3.2. Evaluation of Adhesion to RAJ Explants

**Figure 2:**
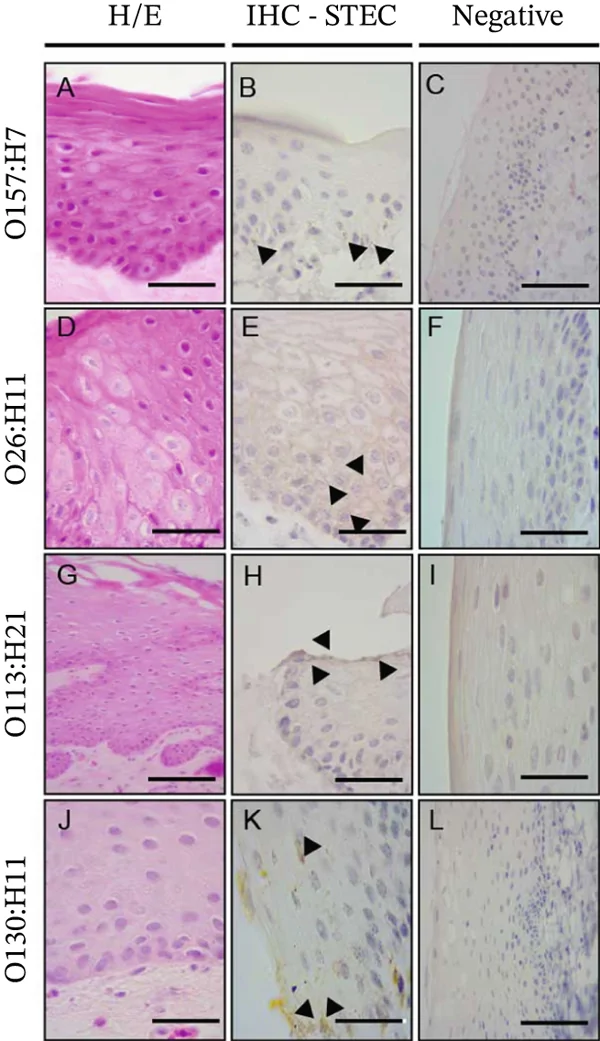
Histological and immunohistochemical evaluation of STEC association with bovine RAJ explants. Representative sections of RAJ explants exposed to O157:H7 (A–B), O26:H11 (D–E), O113:H21 (G–H), and O130:H11 (J–K). The first column (A, D, G, J) shows H/E staining illustrating preservation of the stratified epithelial architecture. The second column (B, E, H, K) shows immunohistochemical detection of STEC, with arrowheads indicating DAB‐positive immunolabeling. The third column (C, F, I, L) shows uninfected control explants, with no detectable DAB immunolabeling. Scale bars = 50 *μ*m.

H/E staining showed that epithelial architecture was well preserved in all explants throughout the experimental period, with clearly defined basal, spinous, and superficial layers, and no evidence of erosion, necrosis, or inflammatory infiltrate in the underlying lamina propria (Figure 2A,D,G,J). Trypan blue staining further supported tissue viability, with no evidence of tissue degradation.

Challenged explants showed moderate to strong perimembranous DAB immunolabeling consistent with bacterial association with the epithelium (Figure 2B,E,H,K), whereas no background or nonspecific signal was observed in uninfected control explants (Figure 2C,F,I,L).

In explants, epithelial association patterns differed between LEE‐positive and LEE‐negative strains. LEE‐positive strains (O157:H7 and O26:H11) showed strong perimembranous immunolabeling extending from the luminal surface into suprabasal and spinous layers, indicating epithelial association beyond the superficial layer. In contrast, LEE‐negative strains (O113:H21 and O130:H11) showed predominantly superficial immunolabeling. Quantitative analysis of DAB% further supported these distinct epithelial association patterns (Figure S1). No significant differences among strains were detected in the apical or middle epithelial regions, whereas significant differences emerged in the basal region, where LEE‐positive strains generally showed greater DAB% than LEE‐negative strains. Overall, these findings indicate that differences among strains became more apparent in deeper epithelial regions, consistent with the broader epithelial association observed histologically for the LEE‐positive strains.

### 3.3. Gene Expression During RAJ Explant Infection

**Figure 3:**
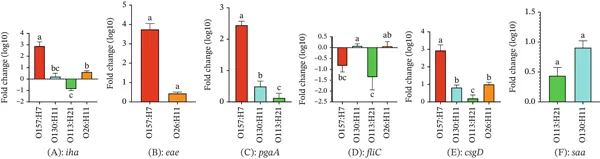
Differential expression of adhesion‐related genes in STEC strains. Expression levels are shown as log10 fold‐change calculated from *ΔΔ*Ct values comparing bacteria recovered from RAJ explants with broth‐grown controls. Bars represent mean ± SD, and different letters indicate statistically significant differences between strains (*p* < 0.05). Genes absent in specific strains were not included.

The relative expression patterns among strains were compared using *ΔΔ*Ct values calculated from normalized Ct data. O157:H7 tended to show the highest relative expression of *eae*, *iha*, *csgD*, and *pgaA*, whereas O113:H21 generally showed lower relative expression levels. In contrast, *fliC* showed divergent patterns, with lower relative expression in O157:H7 and O113:H21 and modest increases in O26:H11 and O130:H11. No differences in *saa* expression were observed between the LEE‐negative strains. Mean ± SD *ΔΔ*Ct and fold‐change values are summarized in Table S4, comparative relative expression is shown in Figure 3, and mean Ct ± SD values for the reference gene across strains and experimental conditions are provided in Table S5.

## 4. Discussion

The bovine RAJ is recognized as a major site of STEC colonization [13]. Although STEC colonization at this site has traditionally been described as a predominantly superficial adherence process, whether this model also applies to LEE‐negative strains remains poorly understood, largely because most studies have focused on LEE‐positive strains. To our knowledge, this is the first comparative study examining early epithelial interaction patterns of LEE‐positive and LEE‐negative STEC strains using both bovine RSE cells and RAJ explants, including field isolates and non‐O157 serotypes. In this study, we analyzed bovine‐origin LEE‐negative strains representing serotypes frequently detected in cattle together with LEE‐positive strains isolated from severe human disease cases.

Overall, the molecular mechanisms underlying STEC adhesion at the bovine RAJ remain incompletely understood, although several adhesins have been associated with epithelial attachment, mainly in studies focused on O157:H7 [29, 30]. In this context, our findings support the existence of strain‐dependent early colonization patterns among epidemiologically relevant circulating isolates, likely reflecting differences in serotype, adhesion‐associated mechanisms, and genomic background [14].

Human epithelial cell lines have been widely used to study STEC adhesion, but they do not reproduce the structure or physiology of bovine tissue [16, 31]. Moreover, LEE‐positive strains display highly specialized interactions with the human intestinal tract [11], which may not accurately reflect colonization dynamics in cattle [10, 32]. In our study, all strains adhered to bovine RSE cells but displayed distinct qualitative attachment patterns. Similar qualitative differences in the absence of marked quantitative variation have been reported previously [16, 33], suggesting that STEC strains may use different strategies for early attachment to bovine epithelial cells. Our findings in O26:H11 and O113:H21 are also consistent with Kudva et al. [34], who described diffuse adherence in these serotypes. Together, these observations support the view that LEE‐encoded factors alone do not fully explain STEC attachment at the bovine RAJ [18]. Similar early adhesion observed in both LEE‐positive and LEE‐negative strains supports the contribution of non‐LEE adhesins during early colonization. Notably, this study provides the first description of O130:H11 adhesion to bovine RSE cells, a serotype associated with severe disease in humans and frequently detected in cattle, extending current knowledge on this serotype.

Assessing attachment in RAJ explants was important to determine whether strain‐dependent patterns observed in RSE cells were maintained in a tissue‐level context that more closely resembles the *in vivo* bovine RAJ. Explants retain the native three‐dimensional architecture, multilayered epithelium, extracellular matrix, and cell–cell interactions, thereby providing a more physiologically relevant environment for pathogen attachment and early colonization events [35, 36]. However, few studies have used bovine RAJ explants to investigate STEC adhesion, and most have relied on a limited number of O157:H7 reference strains [13].

We found distinct adhesion patterns between the analyzed LEE‐positive and LEE‐negative strains in RAJ explants that were not evident in RSE cells, suggesting the presence of strain‐specific epithelial interaction behaviors. In our *ex vivo* model, LEE‐positive strains showed immunolabeling extending beyond the superficial layer into suprabasal and spinous layers, whereas LEE‐negative strains showed a predominantly superficial distribution. Quantitative analysis further supported these distinct patterns, as differences among strains became evident in the basal epithelial region, where LEE‐positive strains generally showed greater immunolabeling than LEE‐negative strains. These findings support distinct epithelial association patterns among the strains analyzed and indicate that preserved epithelial complexity can reveal differences in host–pathogen interaction that are not apparent in simplified cell systems.

To our knowledge, this is the first study to evaluate field STEC strains, including non‐O157:H7 serotypes, in bovine RAJ explants, providing new insights into strain‐dependent epithelial interactions. Although Kudva et al. [13] described only superficial aggregates in RAJ explants using the laboratory O157:H7 EDL933 strain, in our experiments, we observed that LEE‐positive strains showed epithelial association beyond the superficial layer, with immunolabeling extended into suprabasal and spinous layers. First, these findings indicate that field isolates may display interaction patterns not previously captured using reference LEE‐positive strains, such as EDL933. Second, such differences may be relevant to colonization dynamics in cattle, as more extensive epithelial association at the RAJ may contribute to persistent colonization and prolonged fecal shedding. Nevertheless, the extended epithelial association observed for O157:H7 and O26:H11 does not demonstrate intracellular invasion, although eukaryotic cell invasion by STEC has been reported in both human and bovine epithelial models [31, 37, 38].

To explore the contribution of selected adhesion‐related genes to these distinct epithelial association patterns, we examined their expression during RAJ infection. Strain‐dependent transcriptional profiles were observed in response to the bovine RAJ environment, supporting the potential involvement of selected adhesion‐associated genes during early epithelial interaction. Among the LEE‐positive strains, O157:H7 showed the highest relative expression of *eae*, *iha*, *pgaA*, and *csgD*, whereas O26:H11 displayed a similar but less pronounced profile. Iha, a bifunctional adhesin and siderophore receptor, is widely distributed among STEC strains and has been associated with increased adherence in non‐bovine epithelial models [39, 40]. In contrast, *fliC* showed lower relative expression in O157:H7, whereas O26:H11 showed a slight increase. The divergent expression pattern of *fliC* between the two LEE‐positive strains suggests that the contribution of flagellar functions may vary according to serotype and stage of host interaction, which is consistent with previous evidence indicating that H7 flagella are not required for adhesion to bovine RSE cells [41].

Previous studies have suggested that the *eae* gene may not be essential for early attachment at this site [41], but this has only been evaluated in bovine RSE cells using laboratory strains rather than in explants with field isolates. In our explant model, the more extensive epithelial association of LEE‐positive strains suggests that distinct epithelial interaction patterns may be associated with differences in adhesin expression, particularly *eae*. The relevance of this gene is well established, as intimin is a major virulence factor in LEE‐positive STEC and plays a central role in epithelial attachment and attaching‐and‐effacing lesion formation [11]. However, the contribution of *eae* and *iha* has not been previously assessed in bovine RAJ explants, and our findings suggest that these adhesins may contribute to STEC colonization not only in humans but also in cattle.

As described above, LEE‐positive strains showed broader epithelial association within the RAJ, whereas LEE‐negative strains showed predominantly superficial epithelial association, possibly consistent with their more modest transcriptional profiles. These findings suggest that the colonization behavior of LEE‐negative strains may be shaped not only by the absence of the LEE locus but also by a distinct set of adhesion‐related genes, potentially resembling conserved colonization strategies described in commensal *E. coli* [42].

As expected, both O113:H21 and O130:H11 consistently expressed *saa* during RAJ infection. Previous studies identified Saa as an autoagglutinating adhesin that promotes epithelial adherence in LEE‐negative STEC, including increased adhesion to HEp‐2 cells [43, 44]. Our results support its role as an alternative adhesin in the absence of LEE [45]. Despite its proposed role in LEE‐negative pathogenesis, its contribution to bovine colonization has not been previously evaluated in physiologically relevant RAJ models. In contrast to LEE‐positive strains, expression of *iha*, *pgaA*, and *csgD* was lower, suggesting that LEE‐negative strains may rely on a distinct combination of factors for epithelial interaction. Interestingly, O130:H11 showed a slight increase in *fliC* expression, in contrast to what was observed in O157:H7, whereas O113:H21 showed minimal expression. In this context, the consistent expression of *saa* in both strains suggests that it may contribute to their superficial epithelial association and supports its consideration as a potential lineage‐specific target for pre‐harvest intervention.

Despite the novelty of these findings, one limitation of this study is the restricted number of STEC isolates evaluated. The selected strains were chosen to represent epidemiologically relevant isolates from the bovine reservoir and severe human infections, thereby reflecting different points within the STEC transmission chain. Nevertheless, future studies including a broader collection of isolates from different serotypes and host origins will be important to determine the relative contributions of LEE status, genomic background, and strain‐specific characteristics to colonization at the bovine RAJ. A second limitation relates to the interpretation of the RT‐qPCR analyses. Although both the broth‐grown reference cultures and the bacterial inocula used for the explant infections were prepared in the same culture medium, RNA extracted from infected explants contained bacterial RNA derived from tissue‐associated bacteria together with host RNA, whereas the reference cultures consisted exclusively of bacterial RNA. Consequently, differences in the amount of bacterial RNA recovered between broth cultures and tissue‐associated bacteria should be considered when interpreting the relative expression analyses. Therefore, the transcriptional findings should be interpreted as complementary evidence of bacterial responses during RAJ interaction rather than definitive measures of gene regulation. Furthermore, the transcriptional analyses were based on mRNA quantification and therefore do not necessarily reflect protein abundance or functional activity. Consequently, the observed expression profiles should be interpreted as indicators of transcriptional regulation rather than direct evidence of the corresponding phenotypic traits. Likewise, although IHC enabled comparison of bacterial distribution across epithelial layers, it cannot by itself resolve the precise spatial relationship between STEC and epithelial cells. Higher‐resolution imaging approaches will therefore be valuable to further characterize these host–pathogen interactions.

These findings have relevant implications for understanding early STEC pathogenesis at the bovine RAJ and for identifying targets for pre‐harvest control strategies. By showing that naturally circulating strains may display distinct and potentially strain‐specific epithelial interaction patterns, this study expands the current view of early STEC colonization at the bovine RAJ.

## 5. Conclusions

This study provides an integrated evaluation of STEC interaction with the bovine RAJ, showing that the evaluated strains exhibited distinct epithelial association patterns together with strain‐dependent adhesion‐related transcriptional responses during early colonization. These findings suggest that early STEC colonization of the bovine RAJ is influenced by strain‐specific characteristics beyond the simple presence or absence of the LEE island and contribute to a better understanding of STEC colonization of the bovine reservoir and its transmission through the food production chain. Future studies should functionally validate the contribution of candidate adhesins and include a broader collection of isolates from different serotypes and host origins to determine the generalizability of these observations and their potential as targets for pre‐harvest intervention strategies within a One Health framework.

## Author Contributions

Diego Méndez: data curation, formal analysis, investigation, methodology, writing – original draft. José T. Cartajena: Data curation, formal analysis, investigation, methodology, writing – original draft. Jacqueline Flores: formal analysis, investigation. Fernanda Quintanilla: formal analysis, investigation. Jorge Toledo: formal analysis, investigation, methodology, resources, software, writing – original draft. Pablo Cruz: formal analysis, investigation, methodology, resources, software, writing – original draft. Jessica Dorner: data curation, formal analysis, investigation, methodology, writing – original draft. Beatriz Escobar: formal analysis, investigation. María Paz Ríos: formal analysis, investigation. Cristóbal Lavanderos: formal analysis, investigation. Vicente Ferrer: formal analysis, investigation. Paz Rojas: formal analysis, resources. Daniela Luna: data curation, formal analysis, investigation, methodology, writing – original draft. Lisette Lapierre: formal analysis, resources. Indira T. Kudva: investigation, methodology, writing – original draft. Víctor Martínez: conceptualization, investigation, methodology, resources, supervision, validation, visualization, writing – original draft. Nicolás Galarce: conceptualization, funding acquisition, investigation, methodology, resources, supervision, validation, visualization, writing – original draft, writing – review and editing.

## Funding

This study was supported by ANID Fondo Nacional de Desarrollo Científico y Tecnológico (10.13039/501100002850, 1230776) and USDA‐ARS CRIS, 5030‐32000‐225‐00D.

## Ethics Statement

The use of animal tissue was approved by Comité Institucional de Cuidado y Uso de Animales of the Universidad de Chile (permit code 23658–VET–UCH‐e1). The studies were conducted in accordance with the local legislation and institutional requirements. Mention of trade names or commercial products in this article is solely for the purpose of providing specific information and does not imply recommendation or endorsement by the U.S. Department of Agriculture. USDA is an equal opportunity provider and employer.

## Conflicts of Interest

The authors declare no conflicts of interest.

## Supporting Information

Additional supporting information can be found online in the Supporting Information section.

## Supporting information


**Supporting Information 1** Table S1: Phenotypic and genotypic characteristics of selected STEC strains relevant to adhesion. Table S2: Genes used for expression quantification, associated functions, primer sequences, and references. Table S3: Mean number of bacteria attached per cell per serotype. Table S4: Normalized relative expression (*ΔΔ*Ct) values for adhesion‐related genes in STEC strains, calculated from three biological replicates. Table S5: Mean Ct ± SD values of the reference gene *gapA* across STEC strains and experimental conditions.

**Supporting Information 2** Figure S1: Quantitative comparison of DAB‐positive area among STEC strains within apical, middle, and basal regions of bovine RAJ explants.

## Data Availability

The data that support the findings of this study are available from the corresponding author upon reasonable request.
