# Supplementary material for: Distinct Tissue Localization and Transcriptional Profiles of Shiga Toxin‐Producing Escherichia coli With and Without the Locus of Enterocyte Effacement at the Bovine Recto‐Anal Junction
Source: Int J Microbiol. 2026 Aug 29;2026:2576911. doi: 10.1155/ijm/2576911 (PMC13525371; doi:10.1155/ijm/2576911)

Figure S1. Quantitative comparison of DAB-positive area among STEC strains within epithelial regions of bovine RAJ explants. Percentage of DAB-positive area (DAB%) was quantified in the basal, middle, and apical epithelial regions of RAJ explants infected with O113:H21, O130:H11, O26:H11, and O157:H7. Individual points represent analyzed tissue sections. Horizontal lines and error bars represent the median and interquartile range, respectively. Differences in DAB% among STEC strains were evaluated separately within each epithelial region using one-way ANOVA followed by Tukey's multiple-comparisons test. $p < 0.05$; ns, not significant.

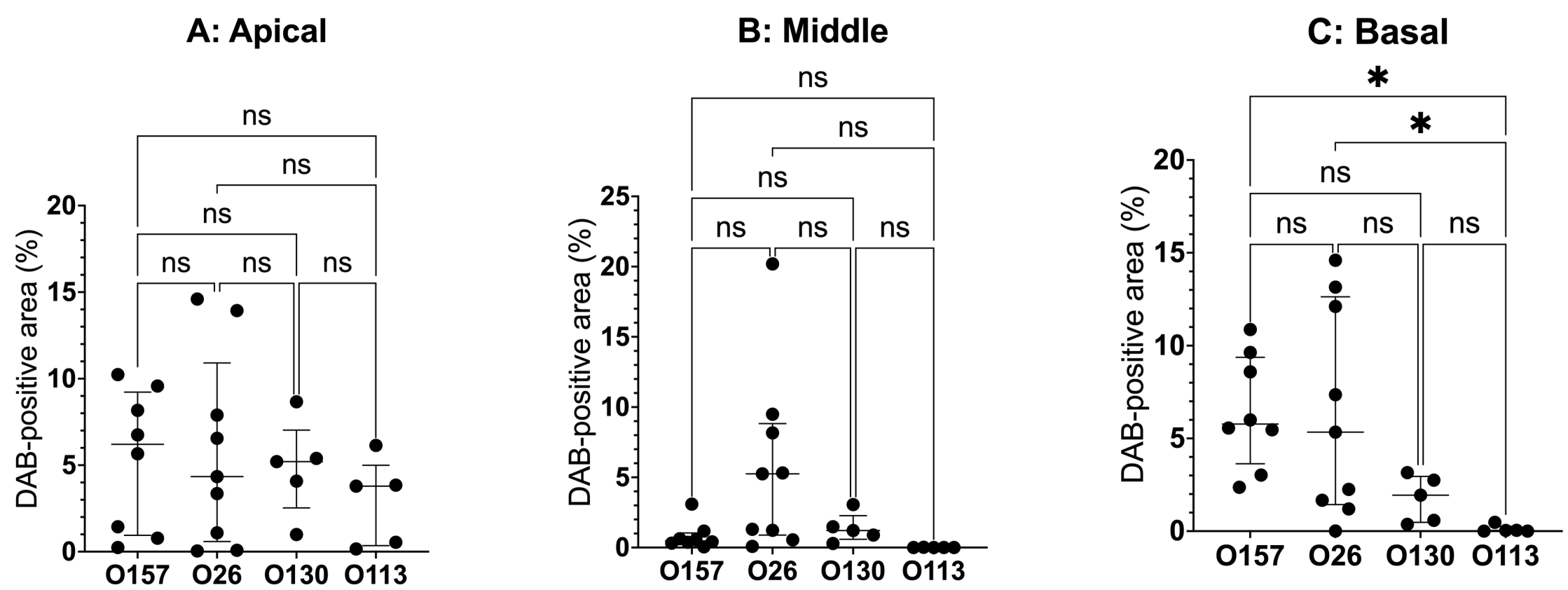

Supplement: Supplementary file 1 [file IJM-2026-2576911-s001.pdf]
